# Current Advances in Immunotherapy for Glioblastoma Multiforme and Future Prospects

**DOI:** 10.7759/cureus.20604

**Published:** 2021-12-22

**Authors:** Selia Chowdhury, Mehedi Hasan Bappy, Santiago Clocchiatti-Tuozzo, Srinidhi Cheeti, Samia Chowdhury, Vraj Patel

**Affiliations:** 1 Internal Medicine, Dhaka Medical College, Dhaka, BGD; 2 Basic Sciences, University of Iowa, Iowa City, USA; 3 Internal Medicine, University of Buenos Aires, Buenos Aires, ARG; 4 Internal Medicine, Chalmeda Anand Rao Institute of Medical Sciences, Telangana, IND; 5 Internal Medicine, Sylhet MAG Osmani Medical College, Sylhet, BGD; 6 Internal Medicine, Smt. Nathiba Hargovandas Lakhmichand (NHL) Municipal Medical College, Ahmedabad, IND

**Keywords:** nanomedicine, car t-cell, oncolytic virus, checkpoint inhibitor therapy, vaccine, immunotherapy, glioblastoma

## Abstract

Glioblastoma is the most frequent and malignant type of brain tumor. It has a reputation for being resistant to current treatments, and the prognosis is still bleak. Immunotherapies have transformed the treatment of a variety of cancers, and they provide great hope for glioblastoma, although they have yet to be successful. The justification for immune targeting of glioblastoma and the obstacles that come with treating these immunosuppressive tumors are reviewed in this paper. Cancer vaccines, oncolytic viruses (OVs), checkpoint blockade medications, adoptive cell transfer (ACT), chimeric antigen receptor (CAR) T-cells, and nanomedicine-based immunotherapies are among the novel immune-targeting therapies researched in glioblastoma. Key clinical trial outcomes and current trials for each method are presented from a clinical standpoint. Finally, constraints, whether biological or due to trial design, are discussed, along with solutions for overcoming them. In glioblastoma, proof of efficacy for immunotherapy approaches has yet to be demonstrated, but our rapidly growing understanding of the disease’s biology and immune microenvironment, as well as the emergence of novel promising combinatorial approaches, may allow researchers to finally meet the medical need for patients with glioblastoma multiforme (GBM).

## Introduction and background

Glioblastoma multiforme (GBM) is the most prevalent type of primary malignant brain tumor in adults, accounting for 60%-70% of gliomas and 15% of main brain tumors [1]. Despite the fact that GBM is a rare tumor with a global incidence of less than 10 per 100,000 people, its dismal prognosis, with a survival rate of 14-15 months following diagnosis, makes it a major public health concern. The World Health Organization (WHO) classification is the current international standard for glioma terminology and diagnosis. It divides gliomas into four grades based on the degree of malignancy assessed by histological criteria. Grade I gliomas are low proliferative potential tumors that can be cured with surgery, but grade II through IV gliomas are extremely aggressive and invasive. Glioblastoma multiforme (GBM) is the most aggressive, invasive, and undifferentiated type of tumor, and it is classified as grade IV by the World Health Organization [2]. The location of the disease and its complicated and varied biology are the main obstacles in GBM therapy. Advances in surgical methods, radiation, and adjuvant chemotherapy have resulted in gradual improvements in the survival and quality of life of patients with GBM, but the prognosis remains bleak [2].

Despite an aggressive standard of care of full surgical resection followed by combined radiation and alkylating chemotherapy, the prognosis remains poor, with a 100% recurrence rate and a median overall survival of about 20 months. Following the success of immune checkpoint inhibitors (ICIs) and oncolytic viral therapies (OVTs) in melanoma and other malignancies, interest in immunotherapy as an alternative method has exploded in recent years [3]. Immunotherapy, which uses the capacity of the host’s immune system to reject cancer cells by generating, augmenting, or reducing immune responses, is quickly becoming a pillar of anticancer therapy. Active immunotherapy involves using tumor vaccines, nonspecific immune stimulants, or cellular vaccines to promote a Th1 immune response, and passive immunotherapy involves injecting effector immune cells into patients to generate an anticancer effect [1]. Despite its success in other malignancies, the ICI inhibition of programmed cell death receptor-1 (PD-1)/programmed death ligand-1 (PD-L1) and cytotoxic T-lymphocyte-associated antigen-4 (CTLA-4) has failed miserably in GBM so far [3]. In patients with recurrent GBM, a phase III trial comparing the PD-1 inhibitor nivolumab to the VEGF-A inhibitor bevacizumab found no difference in survival (CheckMate 143) [4]. Chiocca et al. (2019) have critically assessed a number of viruses that have been reengineered to target GBM [5]. Adenoviruses can be easily modified to cause cell death, promote an immunogenic antitumor response, or transport therapeutic transgenes [3]. G47delta was injected intracranially in combination with adjuvant temozolomide (TMZ) in a phase II trial in recurrent/residual glioblastoma, resulting in an influx of lymphocytes, and interim data showed a 92.3% one-year survival rate [6]. Currently, nanomedicine-based immunotherapies target an immunogenic tumor antigen that crosses the blood-brain barrier (BBB) and either improves or overwhelms the immune system’s ability to regulate itself inside the tumor microenvironment (TME). In the near future, the personalization of GBM immunotherapies with adjuvant therapy combinations such as chemotherapy, radiation therapy, and gene therapy will be pursued [7]. However, because immunotherapy for GBM has had limited or no success, additional research and understanding of this tumor, as well as how to efficiently penetrate cancer cells, is needed.

## Review

Immune evasion mechanisms of glioblastoma multiforme

Besides being the most common primary malignant neoplasm of the central nervous system (CNS) in adults, glioblastoma multiforme (GBM) carries with itself various mechanisms for resisting the host’s immune system. From a unique tumor microenvironment that favors the growth and survival of GBM cells to the usage of immune checkpoint molecules such as PD-L1, CTLA-4, and T-cell Ig and mucin domain 3 (TIM-3), GBM thrives and develops explaining its characteristic high replication. In this section of the review, we will first delve into two of glioblastoma multiforme’s most important immune evasion mechanisms: the control of the tumor microenvironment (TME) to diligently suppress pro-inflammatory leukocytes and the use of checkpoint molecules such as PD-L1 to escape the cytotoxicity of lymphocytes. Later, we will briefly discuss some crucial intrinsic and acquired mechanisms that are of utmost importance when discussing GBM and its immune evasion mechanisms.

Tumor Microenvironment

The cellular environment that surrounds GBM is extremely important for the survival and growth of malignant cells [8,9]. The cells that populate this environment are glioblastoma and glioma stem cells (GSCs), tumor-infiltrating lymphocytes (TILs), myeloid-derived suppressor cells (MDSCs), blood-brain barrier cells (BBBCs), and tumor-associated macrophages (TAMs). Moreover, all of these cells communicate in an intricate manner using secreted paracrine factors, sharing signaling pathways and leading to the increased immune evasion and resistance to treatments that GBM shows.

It was shown that an abundance of GSCs in the tumor microenvironment was more common in high-grade gliomas and that low-grade gliomas had lower quantities of GSCs [10]. Immunosuppressive cytokines and factors such as IL-10, TGF-ꞵ, M-CSF, GM-CSF, versican, and periostin are secreted by the TME, leading to a polarization of GSCs into a tolerogenic subtype, downregulating the immune response. Moreover, M-CSF and GM-CSF promote the migration of macrophages and microglia to the TME and their specialization into the immunosuppressive M2 subtype. The TME has prostaglandin E2 and periostin that promote the passage of TAMs to the M2 subtype; GSCs also secrete factors to benefit this transformation. This subtype of TAM specializes in the secretion of IL-10 and TGF-ꞵ that go on to diminish the pro-inflammatory response.

In comparison to other solid tumors, GBM holds a relatively small amount of TILs compared to GSCs, MDSCs, and TAMs [8]. This is in part explained by the fact that GBM and its TME secrete high amounts of GM-CSF, which in turn acts in the bone marrow to shift hematopoiesis to the generation of granulocytic lineages, and not lymphocytic. However, TILs are an important cell subtype of the TME and must be addressed. They are mostly of the T-regulatory (Treg) cell subtype, as the TIL-induced expression of indoleamine-2,3-dioxygenase (IDO1) in GBM tumor cells recruits more CD25+/FOXP3+ TILs (Treg cells) [8,9].

Another important cell to mention is the myeloid-derived suppressor cells (MDSCs) [11]. These cells differentiate into two subtypes according to their similarities to the mature forms: mononuclear and polymorphonuclear MDSCs. Despite this categorization, MDSCs hold a common purpose: to suppress natural killer (NK), CD8, and CD4 leukocytes. Polymorphonuclear MDSCs do this by secreting reactive oxygen species, secreting anti-inflammatory cytokines, and cross-talking with Treg cells, among other things. On the other hand, mononuclear MDSCs induce several enzymes, such as nitric oxide synthase 2 and arginase that further decrease the activation and proliferation of T-cells.

Thus, it can be seen that the TME is of crucial importance for the tolerogenic properties of GBM. By nesting multiple cell subtypes that secrete anti-inflammatory cytokines and also recruit more immunosuppressive cells, the TME favors the growth of a rapidly dividing and tolerogenic neoplasm.

Usage of Checkpoint Molecules

**Figure 1:**
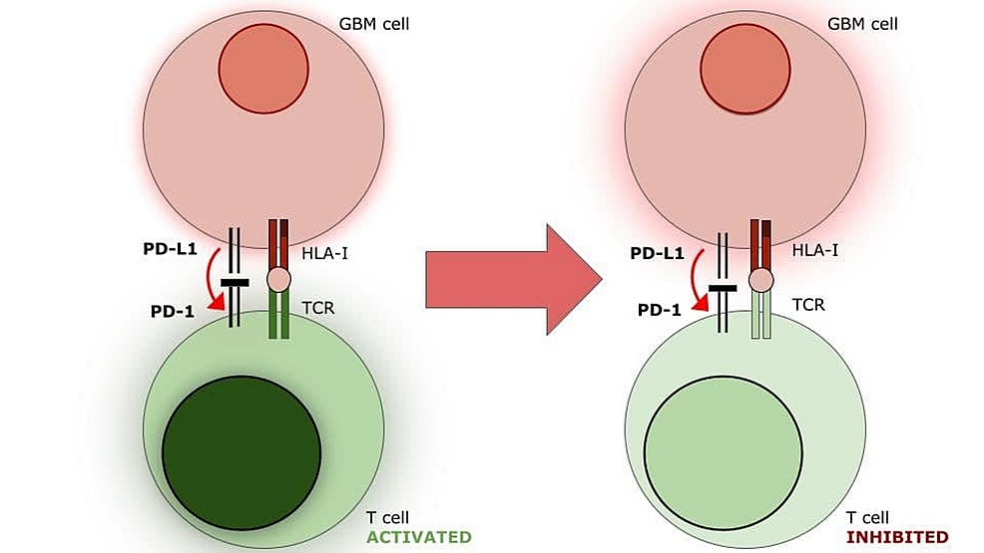
Inactivation of T-lymphocyte through interaction between PD-L1 and PD-1.

The recent discovery of immune checkpoint molecules (PD-1, PD-L1, CTLA-4, TIM-3, etc.) in the biology of tumor immune evasion has revolutionized the world of oncology. We now know that these molecules, which are used normally to fight off autoreactive cells, are used by various cancer cells to suppress the cytotoxic immune response. GBM is no exception to this rule [8,9,12]. Programmed cell death receptor-1 or PD-1 is a receptor that is normally found on activated T-cells. After its binding with the programmed death ligand-1 or PD-L1, a ligand found on GBM, antigen-presenting cells (APCs), NK, and parenchymal cells, the lymphocytes undergo anergy or apoptosis. This mechanism greatly reduces the cytotoxic capacity of the immune system to clear out cancer cells. Besides PD-1, there are other immune checkpoint molecules that work by similar mechanisms. These are the cytotoxic T-lymphocyte-associated antigen-4 (CTLA-4) and T-cell Ig and mucin domain 3 (TIM-3). It is hypothesized that the upregulation of these other immune checkpoint molecules, such as TIM-3, is the basis of the resistance to immune checkpoint inhibitor antibodies such as nivolumab. The combination of dual inhibition, both to PD-1 and TIM-3, is a probable way of bypassing this problem (Figure 1).

Tumor Heterogeneity and Acquired Immunologic Resistance

It is well understood that GBM has other intrinsic as well as acquired immunologic resistance mechanisms besides the ones mentioned in the sections before [12]. One of these intrinsic resistance mechanisms that is important to emphasize is the one understood as “tumor heterogeneity.” GBM is characterized by The Cancer Genome Atlas as having four different molecular and genetic subtypes: proneural, neural, classical, and mesenchymal. The first one, proneural, is defined by having a high expression of the SOX2, OLIG2, and PDGFRA genes, which encode the SOX2, OLIG2 transcription factors, and platelet-derived growth factor-alpha receptor, respectively. The second, neural, expresses neural genes predominantly. The third, classical, has a high amount of mutations in the EGFR gene. Finally, the mesenchymal subtype is identified by mutations in the NF1 gene, which transcribes into the neurofibromin protein. Moreover, it is known that the real biology behind GBM is actually more complex than what this classification can explain. For example, it was shown in a study of 11 GBM tumors, where investigators examined different spatial regions, that different molecular subtypes were present in distinct regions of the same tumor [13]. Thus, the intratumor and intertumor heterogeneity are extensive. This can explain why some antineoplastic treatments deal with one section of the tumor, killing susceptible cells but at the same time selecting more resilient cells for survival. By having a wide variety of molecular differences even in the same tumoral mass, GBM shows a wide variety of different neoantigens as well. This implies that a proper immunologic response has to target quality neoantigens (over quantity) and reassure that these antigens are not only overly expressed throughout the tumor but also that they are absent on normal tissue and that are crucial for the neoplasm’s survival. This results in a new obstacle for an effective immunological response.

An acquired mechanism of defense against the immunologic system that is important to take note of is the one that appears when the neoplasm is confronted with a strong immunological selective pressure. Immune checkpoint inhibitors (e.g., nivolumab) are a growing therapeutic strategy in the battle against cancer such as GBM. Thus, cancer cells from patients treated by these checkpoint inhibitors are immunologically pressured; clones bearing protective mutations will have an advantage over clones that do not mutate correctly to resist the monoclonal antibodies. As an example, Zhao et al. performed a longitudinal study examining 66 patients with recurrent GBM that were treated with PD-1 inhibitors [14]. Using radiographic criteria, they separated patients into “nonresponders” and “responders.” Of these patients, 17 were responders, and it was seen that in the samples of their tumors, cancer cells showed branched evolution patterns. This probably means that there was an elimination of neoepitopes, as GBM cells were constantly pressured by the immune system. Moreover, not only were the responders very few, but the mean difference in survival between responders and nonresponders was also modest, although significant. Responders survived for an average of 14.3 months, while nonresponders survived for 10.1 months. This may be secondary to GBMs’ highly effective acquired immune resistance and evasion against the checkpoint inhibitors.

Current immunotherapeutic strategies for glioblastoma multiforme

Several clinical studies are being conducted in order to develop new treatments for glioblastoma multiforme. Because it is an aggressive brain tumor with poor outcomes even with a long-term treatment strategy, immunotherapeutic modalities are being used to help patients live longer. Radiation, laser therapy, and other therapeutic options work in tandem with immunotherapy to slow the progression of this aggressive malignancy. Antibodies that reeducate tumor macrophages, vaccinations that introduce tumor-specific dendritic cells (DCs), checkpoint molecule inhibition, modified T-cells, and proteins that help T-cells engage directly with tumor cells are among the treatments being explored.

Immune Checkpoint Inhibitors

The two main checkpoints for immunosuppression, programmed cell death receptor-1 (PD-1) and cytotoxic T-lymphocyte-associated antigen-4 (CTLA-4) [15], have recently been the subject of research. Ipilimumab, a CTLA-4 inhibitor, is used in immunotherapy for glioblastoma multiforme. It is a monoclonal antibody against the immunological checkpoint CTLA-4 that prevents it from interacting with B7 [16]. Due to CTLA-4 pathway therapy, there is an increased number of T-regulatory (Treg) cells in glioblastoma multiforme.

Ipilimumab monotherapy is administered in four dosages of 3 mg/kg given over 90 minutes at three-week intervals [17]. Although largely tolerated, the therapy has side effects, including gastrointestinal and skin responses. Seizures, intracranial hypertension, and cerebral edema are all major central nervous system side effects that can be deadly in rare circumstances [18]. Multiple clinical studies have shown that ipilimumab alone is ineffective; hence, it is used in combination with other immunomodulatory drugs as adjuvant therapy. This improves efficacy and reduces the usage of steroids to treat CNS inflammation in patients. Bevacizumab and granulocyte colony-stimulating factors (G-CSF) are two medicines used in combination therapy [19]. The use of temozolomide with ipilimumab to treat newly diagnosed GBM is being investigated in a phase I randomized controlled study. In GBM, monotherapy has a short-term and limited response, but combination therapy has a longer-lasting and enhanced response. The reduction of the tumor microenvironment (TME) of GBM has also been linked to PD-1/PD-L1 checkpoint inhibition. Blocking the PD-1/PD-L1 checkpoint pathway is important for suppressing T-cells, which leads to tumor cell regression [20]. The PD-L1 protein is extensively expressed on the surface of glioblastoma cells, which increases the therapeutic efficacy of blocking this checkpoint pathway. Nivolumab is a monoclonal antibody that targets the PD-1 receptor and disrupts its interaction with PD-1 and PD-L2, resulting in PD-1 pathway-mediated suppression of immune responses, including antitumor responses, and decreased tumor development [21]. In this trial, Omuro et al. found that nivolumab was more effective when given alone rather than in combination [22]. Despite being an effective immunomodulatory method, PD-1/PD-L1 checkpoint pathway blockade faces some challenges, such as the different responses of various genomic subtypes to the blockade, inability to distinguish between primary and recurrent glioblastoma therapy, and which agents should be used in combination if used in combination. To rule out any inconsistencies, more clinical trials are needed to examine the response rate among diverse subgroups.

Chimeric Antigen Receptor T-Cells

Adoptive cell transfer (ACT) using particular T-cells modified to express chimeric antigen receptors (CARs) is another potential strategy for immunotherapy. These are GBM-adoptive T-cell therapies that allow for the selection and introduction of genetically modified cells that are selective for tumor antigens, boosting the proliferation, growth, and maintenance of proper function in order to achieve therapeutic goals [23]. One of the most innovative elements of this strategy is that it adjusts to the surrounding settings of host immunity by removing endogenous lymphocytes that might compete for cytokines with the transplanted cells. Previously, other ACT techniques for GBM treatment, such as natural killer cells or lymphokine-activated assassins, were used, but they did not rely on an HLA-related mechanism of killing. Adoptive cell therapy with autologous tumor-infiltrating lymphocytes, which rely on HLA-restricted tumor antigen identification, has shown considerable clinical regression of GBM. CAR T-cells were shown to be the most efficient of all the ACT-based therapies, but they ran into a serious problem with antigen heterogeneity, which made it difficult to identify antigens expressed on different cancer cells. Intraventricular or intracavitary delivery is possible, with intraventricular potentiate providing greater benefits over side effects than intracavitary. More progress is needed in this area, such as in vivo engraftment, trafficking, persistence, and multiple trials, to make it a viable therapy option for GBM.

EGFRvIII Vaccine

Antitumor vaccinations are another promising immunotherapy technique. It promotes an effective treatment response against GBM by strengthening the adaptive immune system by vaccination. Weller et al. found the efficacy of rindopepimut (also known as CDX-110, vaccination focused at EGFRvIII elimination, which comprises an EGFRvIII-binding peptide incorporated in the keyhole limpet hemocyanin) as conventional chemotherapy for GBM in a randomized control experiment. All of the groups had a 60% reduction in the EGFRvIII gene, according to the study. Patients who received rindopepimut as monotherapy did not have an increase in survival. Seizures, edema, thrombocytopenia, and pulmonary embolism were all reported as serious adverse effects [24]. One of the major drawbacks of this treatment is that not all patients with GBM express EGFRvIII, and only those with certain variations will be eligible for this antitumor vaccine.

Dendritic Cell Vaccines

In several tumors, dendritic cell (DC) vaccines have been offered as a new therapeutic method. They are immunological antigen-presenting cells that express MHC class I and II and play a role in both the innate and adaptive immune systems, making them a prime vehicle as an FDA-approved immunotherapeutic treatment in GBM. Using autologous tumor cell surface peptides as the antigen, Yu et al. studied the DC vaccine. In newly diagnosed high-grade GBM, they reported an improvement in median overall survival [25]. DC vaccination is safe and acceptable; however, immune-related adverse effects such as flu-like symptoms, fever, and skin rashes can occur. Immune tolerance and the activation stage of DC are two potential barriers. The vaccine’s effectiveness may be limited by tumor growth and immune system dysfunction.

Tumor Lysate Vaccines

Tumor lysate is an excellent vaccine antigen for stimulating CD4+ T-cell activity and inducing antitumor immunity via CD8+ T-cells. In their study, Li et al. found that the vaccine treatment and combined treatment groups had longer survival times, lower intracranial tumor volume, and higher immune cell glioma tissue infiltration and IL-2 secretion than the untreated tumor group, indicating that the vaccine is effective in vivo [26]. This vaccine has an excellent clinical efficacy, making it a good clinical application for glioblastoma multiforme.

Oncolytic Viral Therapy

Because of its selectivity and immunostimulatory qualities, oncolytic viral therapy (OVT) is an increasing topic of interest for cancer researchers. Oncolytic viruses are tumor-selective, self-replicating viruses that lyse cancer cells directly. They can be tumor-selective in wild-type or attenuated forms, or they can be manipulated to do so. Through innate, antitumor, and/or antiviral adaptive immunity, they affect the host immune response. Inactivation of antiviral defenses such as type I interferon (IFN) responses in many cancer cells, viral deletions allowing replication only in tumor cells that can substitute for viral defects, tumor-selective uptake via upregulated or mutated receptors, and targeting to tumor promoters are all considered for tumor specificity. Tumor cell death, the creation of endogenous danger signals, the release of tumor-derived cytokines, and direct impacts on innate immune cells are all ways that OVT can modulate the immune system. Its effectiveness is also influenced by the tumor microenvironment (TME) and the blood-brain tumor barrier conditions. GBM is being treated with oncolytic herpes simplex virus (HSV) and low-dose oncolytic adenovirus therapy [27,28]. In GBM, veledimex, an orally administered activator ligand, is suggested. Veledimex is an adenoviral vector (Ad-RTS-hIL-12) that delivers IL-12. It can pass the blood-brain barrier and exert dose-dependent effects [29]. OVT is primarily administered intravenously or intratumorally to give neutralizing effects. The interaction of the innate and adaptive immune systems is important in OVT’s therapeutic response, and combining it with checkpoint inhibitors improves the immunological state for tumor clearance and regression.

Nanomedicine-Based Immunotherapies for GBM

Nanomedicine-based immunotherapies have triggered a systemic and specific immune response against tumor cells, which could be the key to curing cancer that has not been cured. Its efficacy is determined by its capacity to traverse the blood-brain barrier, as well as other factors such as shape, size, functional surface chemistry, circulation half-life, structural stability, and permeability. They are nontoxic and biodegradable, making them a good option for immunotherapy.

Furthermore, CpG (oligodeoxynucleotide-TLR9 ligand expressed by most immune cells) has been demonstrated to stimulate immunological rejection, resulting in long-term protection against gliomas [30]. Antigens are delivered to antigen-presenting cells (APCs) using a synthetic high-density lipoprotein (HDL) nanoparticle. The effect of anti-GBM activity is produced by the intratumoral distribution of docetaxel (DTX) loaded HDL-CpG that mimics nano-disc, greatly enhancing median survival [31]. Antitumor immunity has been found in HDL-CpG loaded with the vaccines ipilimumab (anti-PD-1) and nivolumab (CTLA-4) [32]. In addition, EGFRvIII antigen-loaded hybrid targeted nanoclusters of triblock copolymer MPSDP with zinc oxide (ZnO) core are being developed for intracranial GBM immunotherapy. It secretes immunomodulatory cytokines that stimulate the activation of CD4+ and CD8+ T-cell responses [33].

Clustered regular interspaced short palindromic repeat (CRISPR)/CRISPR-associated protein-9 (Cas9) gene editing is another innovative and adaptable method. Many prokaryotic species have this mechanism, which provides immune protection against bacteria [34]. Scientists use this strategy to boost the cell’s ability to recognize and kill tumor cells in cancer immunotherapies, gene-specific site-specific knockout, and adaptive T-cell engineering.

Obstacles in GBM immunotherapy and how to overcome them

Immunotherapy works by taking the patient’s own immune system and amplifying it or suppressing it for the treatment of a disease. It comes in numerous forms, such as targeted antibodies, cancer vaccines, adoptive cell transfer, tumor-infecting viruses, checkpoint inhibitors, cytokines, and adjuvants. Immunotherapy for GBM has many obstacles to overcome but at the same time has an ample opportunity for growth.

The obstacles that are faced by GBM immunotherapy are numerous. The main obstacle would be the blood-brain barrier. This maintains stability and protects the CNS from the invasion of microorganisms and toxins. It hinders drug delivery by acting as both a physical and biochemical barrier. Also, not all of the BBB is disrupted in GBM, with a significant amount of the tumor being protected by the intact BBB as is seen in MR and PET imaging studies [35]. When a brain tumor such as GBM is present, it can cause degradation of the BBB, causing an increase of vasculature within the tumor and outside it forming the blood-brain tumor barrier [36,37]. This is another barrier that needs to be overcome for intracranial drug transport to occur.

Other than the barrier limitations, the CNS is known to be an immune-privileged site, which means that adaptive immunity and inflammation are highly controlled [38,39]. Recently our understanding of this term has evolved to show that this can be bypassed through the meningeal lymphatic system to some extent and that there is not a total alienation of the CNS from the immune system [40,41]. The brain is also edema-intolerant, which some immunotherapies have the potential to exacerbate [42]. GBM is also known as a “cold tumor,” which is characterized by a lack of T-cell infiltrates within the tumor microenvironment. Immunotherapies are known to be less effective against cold tumors, so our goal should be to convert it into a “hot tumor” [43]. In addition, there seems to be T-cell exhaustion at the tumor site and blood in patients with GBM, contributing to immunosuppression [44].

We can bypass the BBB directly by intracranial convection-enhanced delivery (CED), MRI-guided focused ultrasound (MRIgFUS), intrathecal administration, cell-based targeting strategies, and ligand-mediated strategies [45]. CED is where a catheter is placed at the tumor site, and continuous positive pressure infusion is used to infuse therapeutic agents. This method bypasses the BBB, provides targeted delivery, and enhances interstitial drug distribution [46]. We can still improve on the catheter and image delivery technology as well as optimization of delivery vehicles and transport parameters for this method. The phase 1 trial intracerebral CED of carboplatin in the treatment of recurrent high-grade gliomas showed longer overall survival compared with IV carboplatin [47]. MRIgFUS is a noninvasive technique where focused ultrasound energy waves are used to precisely heat a specific location in the brain. MRI-guided focused ultrasound (MRIgFUS) uses microbubbles that have been administered through IV to cause cavitation that has been reported to disrupt the BBB in small animal models and has been used for the delivery of BBB-impermeable compounds such as a TrkA agonist in this case [48].

Intrathecal administration is a route of administration of drugs directly into the CSF, thus bypassing the BBB. This method of administration is successful for drugs that cannot pass through the BBB [49]. Cell-based targeting strategies include the IV injection of mesenchymal stem cells (MSCs). MSCs are known for their inherent ability to home to sites of injury/brain lesions, migrate large distances, cross the BBB, and communicate with surrounding cells [50]. In one study, human umbilical cord MSCs were intravenously injected in mice, and they were later found in the cerebellum three months after transplantation [51]. The various ligand-mediated strategies constitute ligands targeting transferrin receptors, angiopep-2 peptides, and sugar molecules. These ligands have their respective receptors on the BBB endothelial cells, which enable the transport across the BBB [45].

As we have mentioned above that T-cell exhaustion causes immunosuppression and that CNS is an immune-privileged site where adaptive immunity and inflammation are highly controlled, the following will enhance adaptive immunity by increasing T-cell infiltration and/or enhancing the quality of the T effector cell response in GBM for its treatment. The immunotherapies that will do that are oncolytic viral therapy, DC vaccination, checkpoint inhibition, CAR T-cell therapy, and BiTEs [52]. Oncolytic viral therapy destroys the tumor by activating the immune system. Many different types of oncolytic viruses are being studied, the most common being adenovirus and HSV. There are multiple clinical trials for adult GBM oncolytic viral therapy currently active [53]. Dendritic cell vaccination was shown to increase progression-free survival (PFS) rate in patients with glioblastoma [54].

Checkpoint inhibitors stop the checkpoint and partner proteins from binding together, preventing the “off” signal from being sent, allowing the T-cells to kill the cancer cells. The checkpoint inhibitors can act against the checkpoint proteins CTLA-4 and PD-1. CAR T-cells are a type of treatment where the patient’s T-cells are genetically engineered to attack the cancer cells. The results of CAR T-cells are good, demonstrating feasibility, safety, and efficacy. It still has its own obstacles to overcome, such as the immunosuppressive GBM microenvironment that dampens T-cell proliferation and effector responses [55]. T-cell exhaustion limits the efficacy of checkpoint inhibitors and is taxing for CAR T-cells. By combining both chimeric antigen T-cell therapy and checkpoint blockade, glioblastoma can be treated in two specific approaches with each treatment also mitigating the other’s limitations [56].

Modulation of CNS lymphatic vessels is another method to increase T-cell infiltration. VEGF-C will do this by promoting immune surveillance of the tumor, migration of CD8 T-cells into the tumor, eradication of GBM, and long-lasting antitumor memory response [57]. The latest method of immunotherapy is by using nanoparticles. These nanoparticles have been modified in various ways to be able to easily cross the BBB and treat GBM [58,59]. Some of the ways they can be modified are by changing their size, shape, surface coating, and stability. For example, if we can coat the surface of a nanoparticle with lactoferrin, we can use it to cross the BBB as we know its transporter is expressed on the endothelial cells of the brain [60].

Future Prospects of GBM immunotherapy

Immunotherapy is an effective treatment for several cancers such as head and neck cancer, lung cancer, kidney cancer, bladder cancer, Hodgkin’s lymphoma, and even some brain cancers. Although this has been successful in the treatment of other cancers, it still has a long way to go in the treatment of GBM. Glioblastoma is a life-changing disease, even to date having a dismal prognosis. Although there have been many clinical trials with immunotherapy in GBM, there is still a huge scope for improvement. The reason for this could be due to the fact that we have not completely understood GBM yet, and it is difficult to treat owing to the fact that it is within the CNS and that we have to overcome various obstacles before we get to the root of the problem.

New potential molecular targets have been identified by the genomic profiling of GBM tissue, and the development of novel immunotherapies and neoantigen vaccines to overcome drug resistance is ongoing [61]. One example is the recent trials being conducted using histone modification enzymes and by targeting the epigenetic mechanisms of GBM [62]. Another way is by inhibiting the adipocyte enhancer-binding protein-1 (AEBP1) signaling pathway using ACT001 for the treatment of GBM [63]. In addition, it is better to find more effective animal models to better understand the treatment of GBM, such as the recent discovery of the zebrafish model, as it has a similar brain structure to that of humans [64]. It is also apparent that in order to treat GBM efficiently, combination treatments are required by combining surgery, radiation, chemotherapy, and various immunotherapies together [65,66]. Although we have a huge road ahead, we believe that with more discoveries of the pathophysiology of GBM and how its immune system works, tied together with well-planned out clinical trials, we will be able to eradicate GBM and improve the horrible prognosis and survival rates of the patients.

## Conclusions

Immunotherapy has transformed cancer treatment. Checkpoint inhibitors and CAR T-cells, which have been approved as a first-line treatment for some tumors, have shown spectacular results across a wide range of tumor histology. GBM, on the other hand, has shown high resistance mechanisms against all phases of the antitumor immune response in several investigations. Intrinsic resistance mechanisms and its placement outside the blood-brain barrier prevent the immune response from starting, but adaptive resistance prevents tumor-infiltrating T-cells from infiltrating. Immunotherapy clinical trials in GBM have shown little, if any, effect, with the few individuals who have responded not exhibiting long-term results. A number of factors could account for the unsatisfactory results so far: GBM may have been subjected to extensive immunoediting throughout tumor formation, resulting in a highly immunosuppressive and evasive character. To date, nothing is known about the inherent features of tumors that make them resistant or vulnerable to immunotherapy. Clinical factors such as steroid use, lesion size, and resection status may all play a role in deciding whether or not a patient will react to immunotherapy. Immunotherapy timing could also be crucial. It is possible that administering immunotherapy treatments neoadjuvantly, rather than concurrently with temozolomide and radiation therapy, will induce a more strong immune response. It will also be necessary to look into the molecular factors in the tumor and its microenvironment that need to be targeted or that can predict immunotherapy response. Finally, in order to swiftly obtain information about the potential clinical impact of immunotherapeutic techniques, clinical studies evaluating these approaches must be redesigned. However, current statistics tend to show that combining immunotherapeutic approaches might allow researchers to finally fulfill the medical need for patients with GBM.
